# Circulating Tumor Cells in Patients with Recurrent or Metastatic Head and Neck Carcinoma: Prognostic and Predictive Significance

**DOI:** 10.1371/journal.pone.0103918

**Published:** 2014-08-08

**Authors:** Salvatore Grisanti, Camillo Almici, Francesca Consoli, Michela Buglione, Rosanna Verardi, Andrea Bolzoni-Villaret, Andrea Bianchetti, Chiara Ciccarese, Monica Mangoni, Laura Ferrari, Gianpaolo Biti, Mirella Marini, Vittorio D. Ferrari, Piero Nicolai, Stefano M. Magrini, Alfredo Berruti

**Affiliations:** 1 Medical Oncology Unit, University of Brescia and Spedali Civili Hospital, Brescia, Italy; 2 Department of Transfusion Medicine, Laboratory for Stem Cell Manipulation and Cryopreservation, Spedali Civili Hospital, Brescia, Italy; 3 Radiation Oncology Unit, University of Brescia and Spedali Civili Hospital, Brescia, Italy; 4 Otorhinolaryngology, Head and Neck Surgery Unit, University of Brescia and Spedali Civili Hospital, Brescia, Italy; 5 Department of Radiation Oncology, University of Florence, Florence, Italy; Winship Cancer Institute of Emory University, United States of America

## Abstract

**Introduction:**

We investigated the frequency of detection and the prognostic and predictive significance of circulating tumor cells (CTCs) in patients with recurrent/metastatic (R/M) head and neck carcinoma (HNC) before starting systemic therapy.

**Patients and methods:**

Using the CellSearch technology, CTCs were assessed prospectively in peripheral blood of 53 R/M-HNC patients. We performed spiking experiments to test the diagnostic performance of the CellSearch platform in identifying squamous carcinoma cells.

**Results:**

CTCs were identified in 14 (26%) and 22 (41%) patients at baseline and at any time point, respectively. In univariate analysis ≥2 CTCs had a poorer prognostic role than 0–1 CTC. In multivariate analysis, the presence of one CTC or more was associated with a poor prognosis both in terms of progression-free survival (PFS) [Hazard Ratio (HR): 3.068, 95% confidence interval (CI): 1.53–6.13, *p* 0.002] and overall survival (OS) [HR: 3.0, 95% CI: 1.48–6.0, *p* 0.002]. A disease control after systemic therapy was obtained in 8% of CTC-positive patients as opposed to 45% in CTC-negative ones (*p* 0.03). The epidermal growth factor receptor (EGFR) expression was identified in 45% of CTC-positive patients.

**Discussion:**

In conclusion, CTCs are detected in one out of three patients with RM-HNC. CTC detection is a strong prognostic parameter and may be predictive of treatment efficacy. The frequency of EGFR expression in CTCs seems to be lower than that expected in the primary tumor.

## Introduction

Squamous cell carcinoma of the head and neck (HNC) is the sixth most common cancer worldwide. Concomitant chemo-radiotherapy (CRT) has improved survival and organ preservation in patients with locally advanced disease [1], however treatment failure is observed in more than 50% of cases with stage III–IV tumors. The median survival of patients with persistent, recurrent or metastatic HNC is less than 12 months [2]. Salvage surgery or re-irradiation have a limited benefit at the price of a high incidence of treatment-related morbidity [3]. Systemic chemotherapy including platin-salts agents in combination with infusional fluorouracil and/or a taxane plus/minus the anti-EGFR monoclonal antibody cetuximab is frequently adopted [2]–[4]. Systemic therapy, however, is effective in approximately one third of patients. The characterization of the patient subset destined to have a relatively long survival and obtain benefit from therapy is crucial to tailor individually the best treatment approach and avoid unnecessary side effects. In a retrospective analysis of 390 metastatic patients treated with chemotherapy, Argiris et al. identified a set of five clinic-pathological variables (ECOG performance status, weight loss, location of the primary tumor, prior radiotherapy and tumor cell differentiation) with prognostic significance. This model was also predictive of response to chemotherapy [5], however it is not validated.

Circulating tumor cells (CTCs) can provide meaningful, “realtime” information on the biology and clinical behavior of many tumors [6]–[9]. CTCs are very rare in the blood and, to date, the only standardized and highly reproducible assay, is the CellSearch system (Veridex, Raritan, NJ, USA) a method based on the Epithelial Cell Adhesion Molecule (EpCAM)-specific immunomagnetic separation [10]. The presence of CTCs has already been described in HNC patients, however most studies employed unstandardized systems including immunomagnetic negative separation, flow-cytometry, immunocytochemistry and RT-PCR [11]–[15]. Previous reports of EpCAM expression in carcinomas of the head and neck district showed that EpCAM is overexpressed in approximately 22%–75% of oropharyngeal and larynx carcinomas, in 86% of squamous cell carcinomas of the cervical esophagus and in 83%–100% of salivary glands carcinomas with different immunostaining intensities [16]–[17]. Furthermore, CTCs have been identified by means of the CellSearch in 28%–77% of squamous cell carcinomas of the lung [6], [18].

Our group conducted a multi-center prospective study to verify the presence and clinical utility of CTCs as measured by the CellSearch platform in patients with locally advanced and R/M-HNC. Previously, we reported a correlation between variation of CTCs numbers and response to chemo-radiotherapy in patients with non-metastatic HNC [19]. The current study was undertaken to demonstrate the proportion of R/M patients with detectable CTCs. Secondary aims were to provide information on 1) the prognostic significance of the presence of CTCs in this setting, 2) the role of CTCs detection in predicting treatment response and 3) the frequency of expression of EGFR in CTCs.

## Patients and Methods

### Ethics statement

This diagnostic observational study was conducted at three Oncology Institutions in Italy. The Institutional Ethics Review Board at the Spedali Civili of Brescia (Coordinating Centre) approved the study as part of a wider project of CTC determination in patients with solid neoplasms. However, because of the non interventional nature of the trial and because the patient's clinical management was not influenced by the study results, the other participating centers (Department of Radiotherapy Oncology, University Hospital Careggi of Florence and Department of Radiotherapy Oncology, AUSL-4 of Prato, respectively) approved the study without a formal review by the Ethics Committee Boards. All patients provided a written informed consent for diagnostic and research procedures. The present work was performed in accordance to the ethical principles for medical research involving human subjects of the Helsinki Declaration.

### Study design, patients and treatments

Patient inclusion criteria were a histology-proven diagnosis of squamous carcinoma of the HN region, recurrent or metastatic disease with measurable lesions according to RECIST criteria. Exclusion criteria included poor performance status (ECOG>2) and other concomitant neoplasms. Patients were addressed to either chemotherapy or palliative care when indicated. Chemotherapy was administered as first or subsequent line for recurrent/metastatic disease. Blood for CTC detection was planned to be collected at baseline, after 3 chemotherapy cycles and at treatment completion or progression (PD). Disease status was monitored clinically and by imaging technique at the same time points. Disease control was defined as complete response (CR), partial response (PR) or stable disease (SD). Clinical variables included age, sex, ECOG performance status, weight loss, alcohol, smoking history, histology, grade of differentiation, head and neck primary sub-site, site of relapse, number of metastatic sites, previous treatment and response to chemotherapy. The Argiris prognostic score was also calculated. (Clinical data are accessible in Data S1).

### Blood collection, isolation and enumeration of CTCs

Blood from HN cancer patients was drawn into 10 mL evacuated tubes which contained a cell preservative (CellSave Preservative Tubes, Veridex LLC, Raritan, NJ, USA). Blood from nine normal (ie without previous history or evidence of any neoplasm) individuals as negative controls was also drawn to confirm that CTCs do not circulate in the blood of individuals without cancer. All of them received a clinical examination to confirm absence of either neoplastic or preneoplastic lesions or inflammatory diseases of the HN district at the time of analysis. All CTC analyses were centralized at the Spedali Civili of Brescia and blood samples from peripheral participating centers were sent out by courier. Blood samples were maintained at room temperature and processed for CTC enumeration within 96 hours from collection following the manufacturer's instructions.

### CellSearch assay efficiency of squamous CTCs detection

As the CellSearch system is approved by the US Food and Drug Administration (FDA) for the detection of CTCs from various adenocarcinomas [7]–[9], we sought to verify whether the system was also able to detect cells from squamous carcinomas. In order to estimate the accuracy of the CellSearch System in detecting CTCs in HN neoplasms, we conducted spiking experiments as previously reported [20]. Briefly, three 7.5 mL aliquots of blood from 3 normal blood donors were collected into CellSave Preservative Tubes. The three aliquots from each donor were spiked with different numbers of A-431 cells (ATCC, Rockville, MD, USA), a human squamous cell carcinoma cell line positive for both EpCAM and cytokeratins (Figure S1), to produce separate tubes with 80, 20 and 5 cells per 7.5 mL of blood. Briefly, the human A-431 cell line was cultured in flasks and subsequently harvested by trypsin treatment. Exact concentration of cells per volume (cells/µl) was determined by flow cytometry-based single platform approach using tubes containing pre-determined numbers of microbeads (TruCount, BD Biosciences, San Jose, CA, USA). Analysis was performed using a flow cytometer (FACSCanto II, BD Biosciences, San Jose, CA, USA) by a specific gating strategy in single platform assay. After having determined the exact number of cells present in stock sample, consequent dilution was performed and final concentration of A-431 cells was confirmed applying the same counting strategy. Therefore, 80, 20 and 5 A-431 cells were spiked in triplicate in the Cell Save sample tubes. All tubes were mixed by repeated inversion and allowed to sit overnight at room temperature. On the following day, all the nine tubes were processed and analysed by a single operator according to the manufacturer's instructions. The Pearson correlation analysis was used to establish association between CellSearch-recovered versus spiked CTC counts.

### EGFR analysis on CTCs from R/M-HNC

To measure the percentage of EGFR-positive CTCs from R/M-HNC we used the commercially available CellSearch Tumor Phenotyping Reagent EGFr kit (Veridex LLC, Raritan, NJ, USA) on the fourth channel of fluorescence of the CellSearch system following the manufacturer's instructions. The analytical sensitivity of this kit has not been reported by the manufacturer and we analysed cell lines with different (or absent) levels of expression of EGFR to test the diagnostic performance of the procedure. The CellSearch was able to correctly detect all the spiked cells from the three EGFR-positive cell lines as EGFR-positive and correctly identified the EGFR-negative cell line as EGFR-negative. (data not shown).

### Statistical analysis

The primary objective of the study was to assess the frequency of CTCs in the peripheral blood of patients with RM-HNC measured by the CellSearch system. The sample size was calculated in order to reject the null hypothesis that the proportion of patients with detectable CTC was less than 5% and to provide a statistical power of 80% to demonstrate that at least 35% of patients had at least 1 CTC/7.5 mL. With a two-sided alpha of 0.05, 50 consecutive patients meeting the inclusion criteria were planned to be enrolled. Secondary objectives were to correlate the presence of CTCs with outcome endpoints and response to chemotherapy.

The relationship between categorical covariates was assessed with a Fisher's exact test. PFS and OS were defined as the time elapsed from baseline CTC analysis to the first evidence of disease progression and death or last follow-up, respectively. Survival curves were calculated with the Kaplan Meier method and compared with the log-rank (Mantel-Cox) test. Cox proportional hazard model was employed to assess the HR of progression and death both in uni- and multivariate analyses. All tests were two-sided and the p<.05 was considered statistically significant. Statistical analyses were conducted with the SPSS software (SPSS version 17.0, Chicago, IL, USA). The REporting of tumor MARKer Studies (REMARKS) guidelines were followed in reporting results of this study [21].

## Results

### Patient demographics

From January 2009 to February 2011, 57 consecutive patients were enrolled in the study. Four patients were not evaluable, two because of technical defaults of the CellSearch system and two because of lack of clinical information. The characteristics of the final 53 evaluable patients are outlined in Table 1. The vast majority of patients had a squamous cell carcinoma. Four patients had an undifferentiated carcinoma of the nasopharynx. Fifteen (28%) patients were metastatic at diagnosis and 38 had recurrent disease. Forty patients (75%) were chemotherapy-naïve. Chemotherapy was administered to 45/53 (85%) patients while 8 patients with severe comorbidities received best supportive care. Chemotherapy consisted of cisplatin monotherapy in 1 patient (2%), cisplatin plus fluorouracil in 16 (36%) patients, cisplatin, fluorouracil and paclitaxel in 21 (46%) patients, cisplatin and cetuximab in 1 patient (2%), methotrexate in 4 (9%) patients and vinorelbine in 2 (4%) patients. In the group of nine individuals selected as negative controls, median age was 51 years, 6 were males and 3 were females.

**Table 1:** Patients characteristics at baseline.

		Evaluable patients (*n* = 53)
Characteristic		*n* (%)
Age (years)	Median	59
	Range	20–79
	<70 years	42 (79)
	≥70 years	11 (21)
Sex	Male	42 (79)
	Female	11 (21)
Baseline ECOG performance status	0	12 (23)
	1	27 (51)
	2	13 (24)
	3	1 (2)
N. of Argiris factors	0–2	14 (26)
	≥3	39 (74)
Weight loss	<5%	29 (55)
	≥5%	24 (45)
Alcohol abuse	Yes	26 (49)
	No	27 (51)
Smoking history	Yes	46 (87)
	No	7 (13)
Anatomic distribution of primary tumor	Nasopharynx	4 (8)
	Nasal sinus	4 (8)
	Oral cavity	17 (32)
	Oropharynx	7 (13)
	Hypopharynx	4 (8)
	Larynx	15 (28)
	Cervical esophagus	2 (3)
Tumor grade	1–2	44 (83)
	3	9 (17)
Disease extent	Locally advanced	26 (49)
	Metastatic	27 (51)
No of disease sites	1	30 (56)
	2	20 (38)
	3	2 (4)
	4	1 (2)
Prior treatments for primary tumor	Surgery	32 (60)
	Radiotherapy	34 (64)
	Chemotherapy	15 (28)
N. of prior chemotherapy lines for metastatic disease	0	40 (75)
	1	7 (13)
	2	1 (2)
	3	5 (10)

### CellSearch assay efficiency of squamous CTCs detection

**Figure 1:**
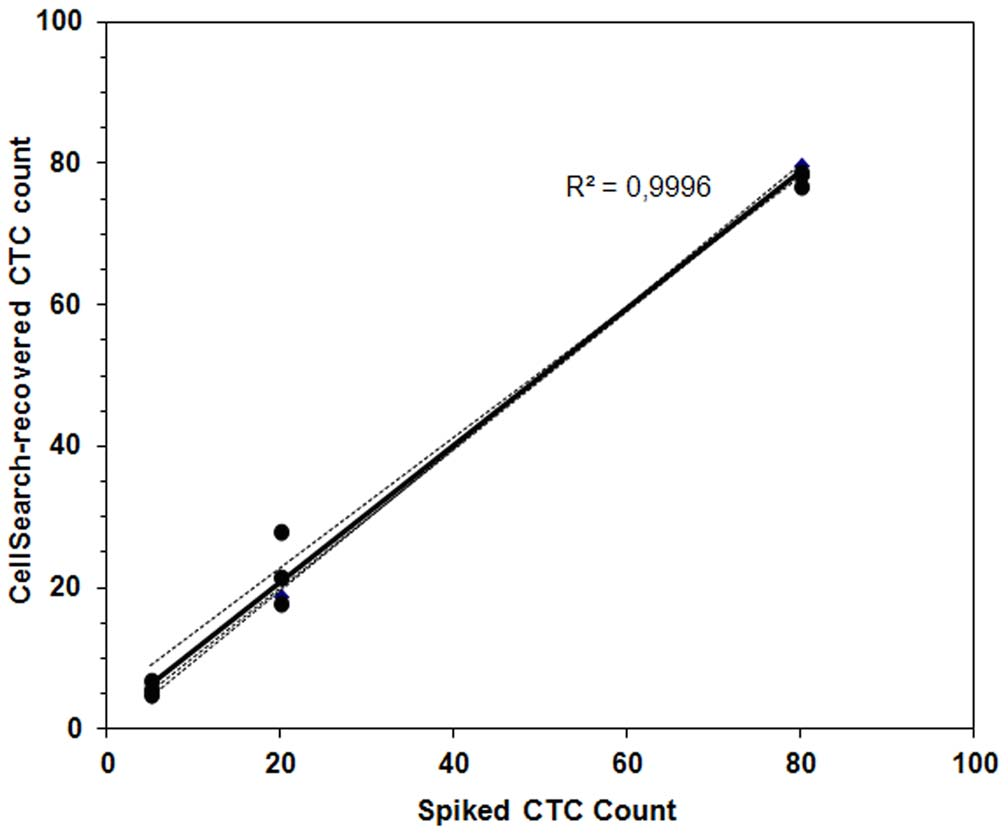
Recovery efficiency of known numbers of spiked A-431 cells from 7.5 mL of blood. The number of spiked cells is plotted against cells recovered by the CellSearch. The Pearson correlation value (R^2^) indicates strong correlation between spiked and recovered cells.

The approximate number of A-431 cells spiked into the blood of the three healthy donors was plotted against the number of the CellSearch-recovered cells in the samples and regression analysis using the number of observed CTCs versus the number of expected CTCs resulted in a best fit line with a slope of 1.03 (95%CI: 0.77–1.3), an intercept of −1.57 (95%CI:−13.9–10.8), and a correlation coefficient (R^2^) of 0,9996 (Figure 1). The assay efficiency of recovery was >90%, indicating that the platform was able to detect squamous cells.

### CTCs assessment

Thirty-seven patients had CTC assessed at least at two time points while sixteen patients had only one sample evaluated. Twenty-two (41%) patients had detectable CTCs at least at one time point during clinical observation. In patients with detectable CTCs, median and mean CTCs values were 1 and 5 (±10 SD), respectively. We therefore used 1 CTC/7.5 mL as cutoff for subsequent analyses. At baseline, 14 (26%) patients had at least 1 CTC detected. Nine of them (64%) had 1 CTC, two (14%) had 2–10 CTCs and three (20%) more than 10 CTCs (Table 2). None of the nine normal volunteers had detectable CTCs. At univariate analysis the presence of CTCs at baseline was not related to any of the clinical variables considered. (Table 3).

**Table 2:** CTCs prevalence in recurrent/metastatic head and neck cancer patients.

Characteristic		*n* (%)
Evaluable patients		53 (100)
CTCs+ pts at baseline		14 (26)
CTCs numbers at baseline	Median CTCs	1
	Mean CTCs ± SD	5±10
	Range	1–43
CTCs+ pts at anytime		22 (41)
N. of CTCs determinations	1	16 (31)
	2	21 (39)
	3	5 (9)
	4	11 (21)

**Table 3:** Univariate associations between CTCs at baseline and clinico-pathologic characteristics.

Characteristic		N. pts with ≥1CTC at baseline/total (%)	Fisher's exact test *p*
Age (years)	<70 years	13/42 (31)	.251
	≥70 years	1/11 (9)	
Sex	Male	12/42 (28)	.706
	Female	2/11 (18)	
Baseline ECOG PS	0	5/23 (22)	.547
	≥1	9/30 (30)	
Weight loss	<5%	7/29 (24)	.760
	≥5%	7/24 (29)	
Alcohol abuse	Yes	8/26 (31)	.544
	No	6/27 (22)	
Smoking history	Yes	13/46 (28)	.660
	No	1/7 (14)	
Main HN anatomical grouping	Upper HN (nasoph+nasal sinus)	2/8 (25)	.916
	Mid HN (oroph+oral cavity)	7/24 (29)	
	Lower HN (larynx+hypoph+esoph)	5/21 (24)	
Tumor grade	1–2	14/44 (32)	.092
	3	0/9 (0)	
Relapse/recurrent disease	Local	6/26 (23)	.757
	Distant	8/27 (30)	
No of sites of metastasis	1	5/30 (16)	.115
	>1	9/23 (39)	
M+ de novo vs relapsed	De novo	3/15 (20)	.732
	Relapsed	11/38 (29)	
Prior surgery for primary tumor	Yes	8/31 (26)	1.0
	No	6/22 (27)	
Prior RT for primary tumor	Yes	9/36 (25)	.748
	No	5/17 (29)	
Prior CT for primary tumor (concomitant/neoadjuvant)	Yes	4/25 (16)	.225
	No	10/28 (36)	
N. of prior CT lines for metastatic disease	0	10/40 (25)	.725
	≥1	4/13 (31)	
N. of Argiris factors	0–2	3/14 (21)	.735
	≥3	11/39 (28)	

### Prognostic significance of CTCs in HNC patients

**Figure 2:**
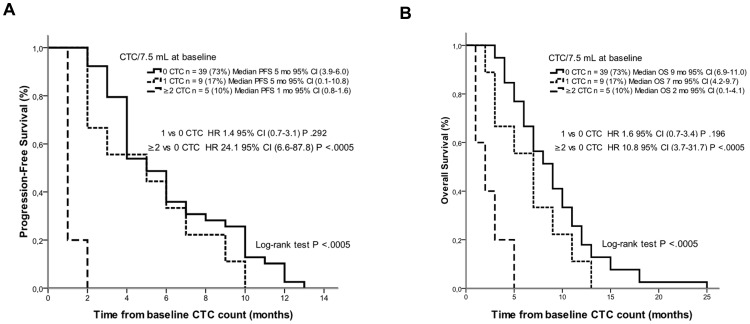
Kaplan-Meier curves of (A) progression-free survival and (B) overall survival in patients with 0, 1 and ≥2 CTCs.

At the date of last follow-up (March 2012) all patients had progressed and died. In the overall population median PFS and OS from CTC analysis were 4 and 7 months, respectively. Median PFS in patients with 0, 1 and ≥2 CTCs at baseline were 5 months (95% CI 3.9–6.0), 5 months (95% CI 0.1–10.8) and 1 month (95% CI 0.8–1.6), respectively, (log-rank test *p*<.0005). Median OS in patients with 0, 1 and ≥2 CTCs at baseline were 9 months (95% CI 6.9–11.0), 7 months (95% CI 4.2–9.7) and 2 months (95% CI 0.1–4.1) respectively, (log-rank test *p*<.0005) (Figure 2A and 2B). These results indicate that ≥2 CTCs rather than 1 CTC might be a better cutoff indicator for both PFS and OS.

**Figure 3:**
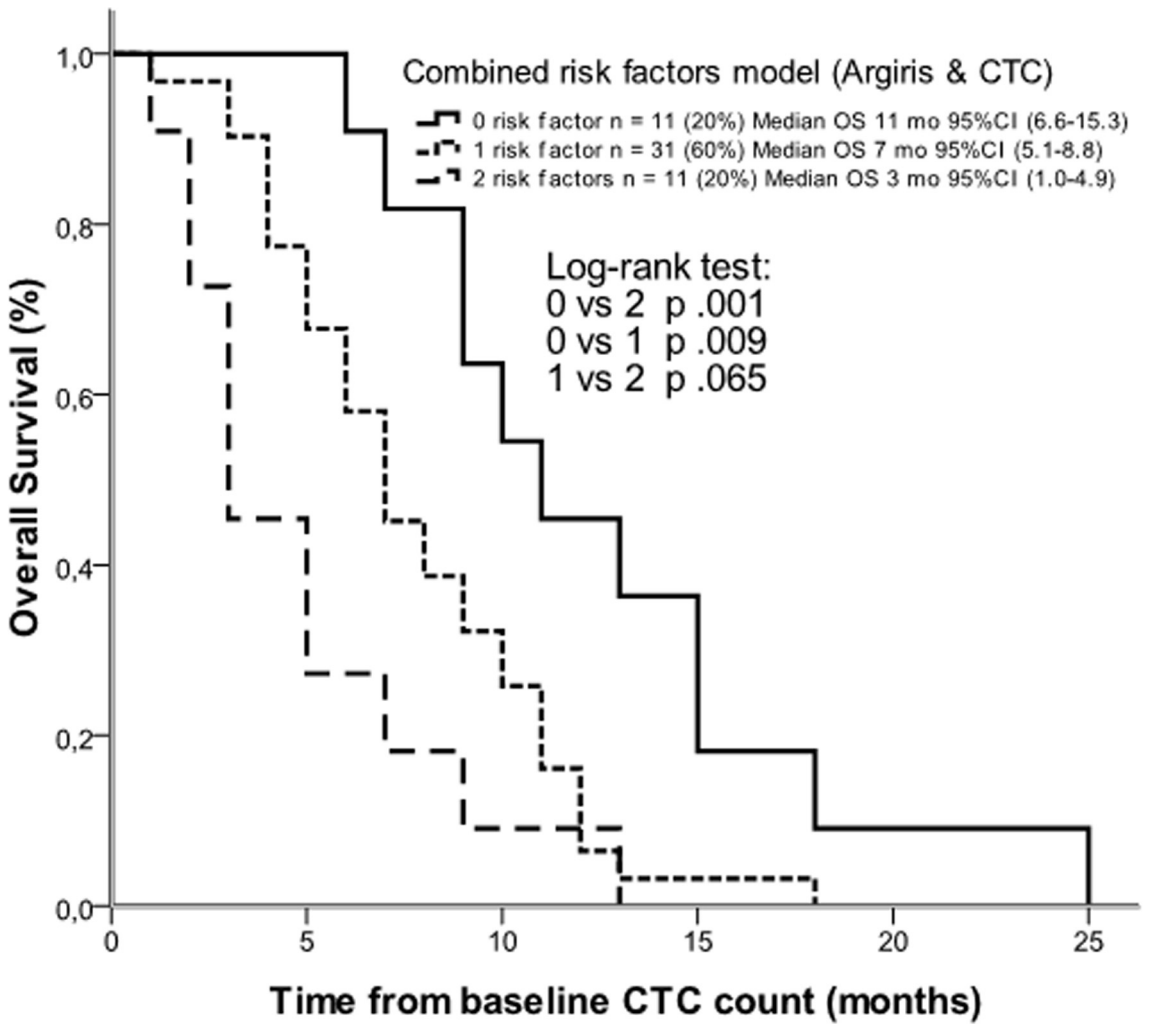
Kaplan-Meier estimates of overall survival according to a combined risk factors model with Argiris factors and CTCs. Continuous line indicates absence of both risk factors; small dotted line indicates the presence of only one of the two risk factors; large dotted line indicates the presence of both risk factors.

Combining Argiris factors with CTC results, overall survival was better in patients with low Argiris risk and no CTC (median 11 months, 95% CI 6.6–15.3) than in patients with high Argiris risk and at least 1 CTC (median 3 months, 95% CI 1.0–4.9) (log-rank test *p* .001) (HR .22, 95% CI .09–.54). Patients with one of the 2 risk factors had an intermediate survival (median 7 months, 95% CI 5.1–8.8) (HR .52, 95% CI .26–1.0) (Figure 3).

At univariate analysis, variables with statistically significant impact on PFS and OS were the site of relapse (local vs distant) (*p* .015 and *p* .011, respectively), the Argiris prognostic factors (≥3 vs 0–2) (calculated only for OS, *p* .017) and CTCs at baseline (Table S1). At multivariate regression analysis, the covariates that maintained an independent prognostic significance both for PFS and for OS were CTCs at baseline (HR 3.0, 95% CI 1.53–6.13, *p* .002 and HR 3.0, 95% CI 1.48–6.0, *p* .002) and the site of relapse (HR 2.68, 95% CI 1.44–4.97, *p* .002 and HR 2.34, 95% CI 1.14–4.8, *p* .019), respectively (Table 4).

**Table 4:** Multivariate analysis of progression-free and overall survival.

Characteristic		PFS			OS		
		HR	95% CI	*p*	HR	95% CI	*p*
CTCs at baseline	≥1 vs 0	3.0	1.53–6.13	.002	3.0	1.48–6.02	.002
Relapse/recurrent disease	Local vs Distant	2.7	1.45–4.98	.002	2.3	1.14–4.80	.019
Argiris prognostic factors^1^	≥3 vs 0–2	-	-	-	1.3	.60–2.89	.427

1 Argiris factors were included in the overall survival model only. Abbreviations: PFS, progression-free survival; OS, overall survival; HR, hazard ratio; CI, confidence intervals; CTCs, circulating tumor cells.

### CTCs at baseline as predictors of response to chemotherapy and variation of CTCs number after chemotherapy

**Figure 4:**
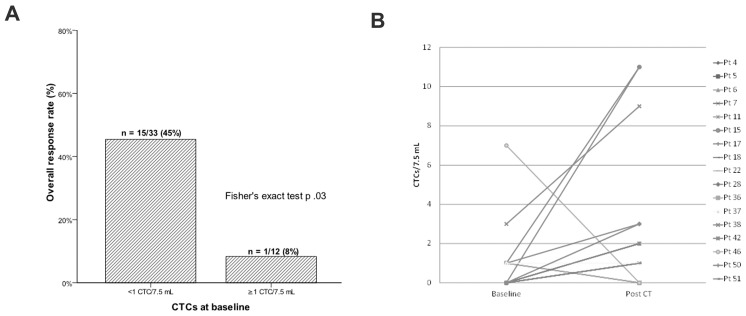
Association between the presence of CTCs before starting a new line of chemotherapy and response to treatment. Higher response rate is observed in CTC-negative patients at baseline (A). Dynamic variation of CTCs numbers before and after treatment in patients (n = 10) with at least two determinations and at least one CTC at any time point. CTCs changes did not correlate with tumor response (B).

Response to chemotherapy was evaluated in 45 treated patients after 3 cycles and at treatment completion or progression. Sixteen (35%) patients obtained disease control (9 PR and 7 SD) and the remaining 29 (65%) had PD. The clinical control of the disease was mainly confined in patients with 0 CTC (15/33, 45%) while only one (8%) PR was observed in the 12 patients with detectable CTCs at baseline (p .03) (Figure 4A).

**Figure 5:**
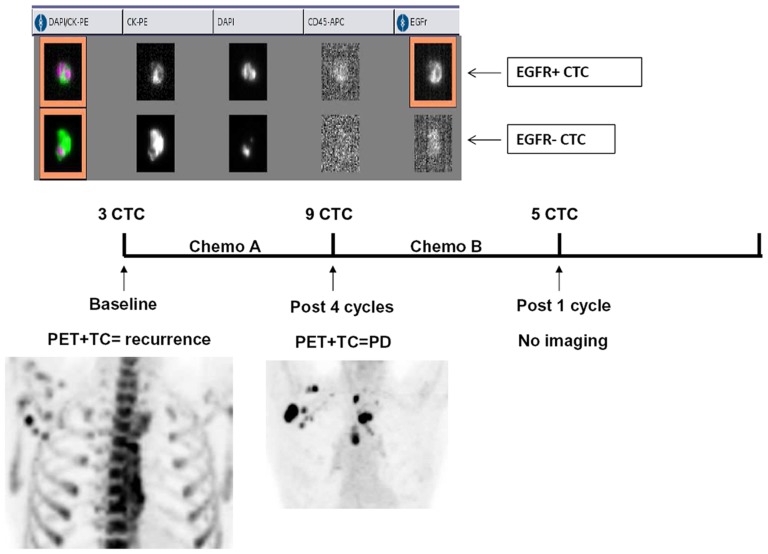
Example of CTCs analysis in a patient with mediastinal and axillary nodal metastases from an oropharyngeal squamous cell carcinoma. (A) the CellSearch output of baseline CTC analysis showing two CTCs with heterogeneous EGFR expression. (B) Timeline of CTC analysis and treatments. (C) Correlative imaging analysis by CT/PET at baseline and after chemotherapy. In this patient 3 CTCs were detected at baseline. After 4 cycles of a chemotherapy, CTC number rised to 9 suggesting progressive disease then confirmed by CT/PET imaging.

Ten out of the 14 CTC-positive patients at baseline were tested before and after chemotherapy. Of them, five had a increase, one remained stable and four had a decrease of CTCs after treatment. By contrast, seven patients without CTCs at baseline had detectable CTCs at the end of treatment (Figure 4B). As an example, Figure 5 illustrates the outcome in terms of CTCs and imaging of a patient progressing after a line of chemotherapy. Changes in CTCs levels were not found to be correlated with tumor response.

### EGFR characterization of CTCs in HNSCC patients

The EGFR expression was assessed in 41 samples. Of the 22 patients with at least one CTC during clinical observation, 10 (45%) had CTCs with detectable levels of EGFR as measured with the CellSearch system and 12 (55%) had EGFR-negative CTCs. Of note, consistent heterogeneity in EGFR expression was observed intra- and intersample (Figure 5). On average, when more than one CTC was counted in 7.5 mL of blood, EGFR was expressed in 25% of the detected CTCs within the same sample. Furthermore, in 8 patients with EGFR-positive CTCs evaluated at two or more time points, EGFR was detected in 37% of the samples indicating that EGFR expression is modulated over time within the same patient.

## Discussion

This study demonstrated that 26% of R/M-HNC patients at baseline conditions and 41% at any time point had ≥1 CTC. The spiking experiments performed with the EpCAM-positive cell line A-431 confirmed that the CellSearch technology can detect squamous CTCs in blood. These results are in line with recent experiences in which 16%–40% of stage III–IV HNC patients had 0–2 CTCs by the CellSearch [22], [23].

The low median numbers of CTCs detected in HNC patients by the CellSearch test could reflect a true low frequency of CTCs in HNC or may be the result of an intrinsic low expression of the capture antigen, EpCAM molecule, on cancer cells surface. Alternatively it suggests the existence of epithelial-to-mesenchymal transition (EMT) phenomena with a higher number of circulating cancer cells lacking epithelial markers such as EpCAM or cytokeratins [24], [25].

There is an urgent need of prognostic and predictive factors in recurrent/metastatic-HNC patients and our results showed that CTCs could serve as new independent prognostic parameter for both PFS and OS. In univariate analysis >2 CTCs had a poorer prognostic role than 0–1 CTC in patients before starting a new chemotherapy. However, this effect was observed also at any time point during clinical observation (data not shown).

The largest analysis of prognostic factors in recurrent and metastatic HNC, published by Argiris et al, identified a set of clinico-pathological factors that correlated with the patient outcome [5]. Noteworthy, in our study, after adjustment for the presence of CTCs, the Argiris criteria were no longer prognostic. By combining Argiris high risk criteria with the presence of CTCs we found a good separation in terms of survival among patients with both risk factors, one risk or none of the two. These data confirm the emerging evidence that the presence of cancer cells in the blood is *per se* associated with more aggressive features of the neoplasm, independently of the patient characteristics and the tumor stage. Another interesting aspect of our study was that there was no difference in terms of CTCs between patients with local recurrence or distant metastases. This result suggests that HNC, traditionally considered a disease with local tropism, is indeed characterized by circulation and re-circulation of cancer cells via the bloodstream regardless of the metastatic site, local or distant [26].

In our series the presence of CTCs was also predictive of chemotherapy activity. The clinical control of the disease, in fact, was mainly confined in patients with 0 CTC as opposed to their counterpart. Again, it could be hypothesized that those cancer cells that enter into the blood have acquired more aggressive features than resident cancer cells, including enhanced survival and chemoresistance [27]. Thus, the presence of CTCs before starting a new chemotherapy or their appearance during treatment could identify poor-responder patients. This interesting observation deserves confirmation.

CTCs offer the unique opportunity to monitor the changes in tumor biology over time. EGFR is overexpressed in over 90% of HNC [28] although with a wide range of expression [29]. In this work, the CellSearch was able to identify EGFR signals in 45% of patients with CTCs at any time point. These data suggest a huge discordance of EGFR expression between the primary tumor and CTCs and this could indicate a modulation of EGFR expression over time. Similar findings were already described in metastatic breast cancer patients in whom a discordance rate of 42% in terms of Her2-positivity between primary breast cancer and CTCs was observed [30]. Again, given that EMT processes induce downregulation of epithelial markers and that EMT facilitates intravasation of cancer cells within the bloodstream [31], the presence of EGFR-negative CTCs could identify cancer cells with more proclivity to metastasize.

EGFR determination by immunohistochemistry (IHC), either in the primary or in metastatic lesions, is not required for Cetuximab prescription according to both FDA and EMEA guidelines. In the Extreme trial, response rate in the Cetuximab arm was 36%. However, neither EGFR expression by IHC nor EGFR gene copy number by FISH in the primary tumor were found to be predictive of response in two retrospective analyses [32], [33]. Based on our results, evaluation of EGFR expression on CTCs could be prospectively analyzed in Cetuximab-treated patients to establish possible predictive significance.

In conclusion, this paper demonstrates for the first time that CTCs can be detected in one third of patients with RM-HNC and their presence strongly correlates with unfavorable prognosis independently of tumor and patient's characteristics. Our data also suggest a role of CTCs in predicting the treatment activity, although these results must be interpreted with caution. The clinical and therapeutic implications of CTCs in HNC and their molecular heterogeneity deserve to be further studied.

## Supporting Information

Figure S1 **Phenotype analysis of the A-431 squamous cell line in terms of EpCAM, cyokeratins and EGFR expression.**
(TIF)

Table S1 **Univariate analysis of progression-free and overall survival.**
(DOCX)

Data S1 (SAV)
