# Supplementary figures and images for: Circulating Tumor Cells in Patients with Recurrent or Metastatic Head and Neck Carcinoma: Prognostic and Predictive Significance
Source: PLoS One. 2014 Aug 8;9(8):e103918. doi: 10.1371/journal.pone.0103918 (PMC4126745; doi:10.1371/journal.pone.0103918)

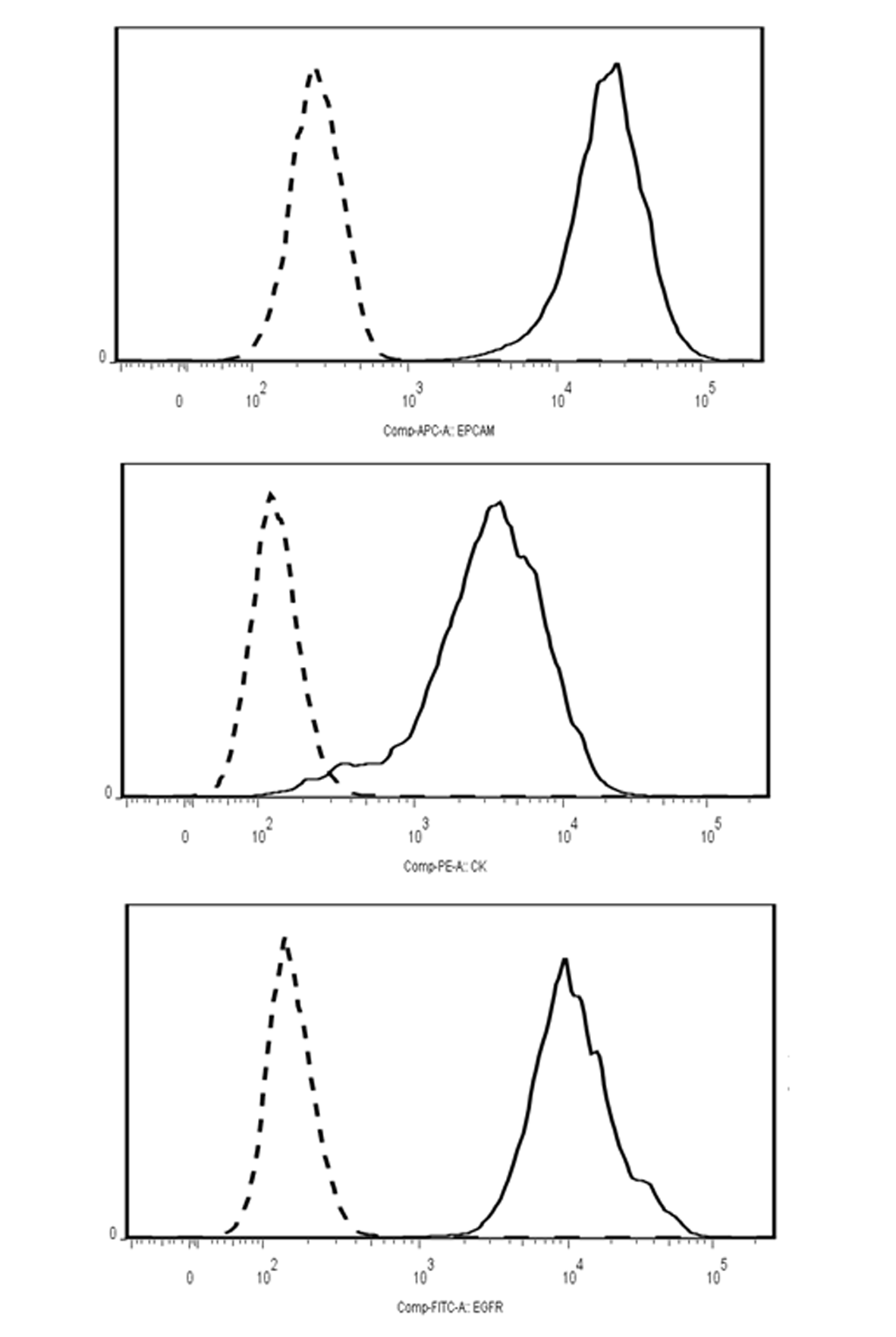

Supplement: Figure S1 — Phenotype analysis of the A-431 squamous cell line in terms of EpCAM, cyokeratins and EGFR expression. (TIF) [file pone.0103918.s001.tif]
