# Supplementary material for: Circulating Tumor Cells in Patients with Recurrent or Metastatic Head and Neck Carcinoma: Prognostic and Predictive Significance
Source: PLoS One. 2014 Aug 8;9(8):e103918. doi: 10.1371/journal.pone.0103918 (PMC4126745; doi:10.1371/journal.pone.0103918)
Supplement: Table S1 — Univariate analysis of progression-free and overall survival. (DOCX) [file pone.0103918.s002.docx]

**Table S1**. Univariate analysis of progression-free and overall survival

| Characteristic | | PFS | | | | OS | | | |
| --- | --- | --- | --- | --- | --- | --- | --- | --- | --- |
|  |  | Median (mo) | HR | 95%CI | p | Median (mo) | HR | 95%CI | p |
| Age (years) | <70 vs ≥70 years | 4 vs 6 | 1.37 | .699-2.707 | .355 | 7 vs 8 | 1.73 | .852-3.536 | .129 |
| Sex | Female vs Male | 3 vs 5 | 1.74 | .884-3.449 | .108 | 5 vs 8 | 1.96 | .980-3.950 | .057 |
| Baseline ECOG PS | ≥1 vs 0 | 4 vs 5 | 1.08 | .624-1.886 | .773 | 6 vs 8 | 1.05 | .793-1.391 | .733 |
| Weight loss | ≥5% vs <5% | 4 vs 5 | 1.19 | .685-2.082 | .531 | 7 vs 9 | 1.54 | .877-2.729 | .132 |
| Alcohol abuse | Yes vs No | 4 vs 6 | 1.31 | .758-2.285 | .329 | 7 vs 7 | 1.29 | .746-2.249 | .358 |
| Smoking history | No vs Yes | 4 vs 6 | 1.11 | .746-1.658 | .603 | 7 vs 7 | 1.09 | .715-1.689 | .668 |
| Tumor grade | 1-2 vs 3 | 4 vs 6 | 1.47 | .683-3.469 | .324 | 7 vs 9 | 1.24 | .604-2.566 | .552 |
| Relapse/recurrent disease | Local vs Distant | 4 vs 7 | 2.00 | 1.143-3.54 | .015 | 5 vs 10 | 2.0 | 1.185-3.68 | .011 |
| No of metastatic sites | 1 vs 2-4 | 4 vs 5 | 1.00 | .564-1.787 | .990 | 7 vs 7 | 1.18 | .678-2.0 | .555 |
| Prior surgery for primary tumor | Yes vs No | 5 vs 4 | 1.24 | .705-2.193 | .452 | 7vs 9 | 1.09 | .629-1.911 | . 745 |
| Prior RT for primary tumor | Yes vs No | 4 vs 4 | 1.02 | .569-1.827 | .948 | 7 vs 7 | 1.22 | .671-2.235 | .510 |
| Prior CT for primary tumor | Yes vs No | 5 vs 4 | .876 | .507-1.514 | .636 | 8 vs 7 | .938 | .543-1.623 | .820 |
| N. of prior CT lines for metastatic disease | ≥1 vs 0 | 4 vs 4 | .873 | .454-1.677 | .683 | 9 vs 7 | .729 | .378-1.406 | .345 |
| Argiris prognostic factors^1^ | ≥3vs 0-2 | - | - | - | - | 6 vs 10 | 2.2 | 1.15-4.23 | .017 |
| CTCs at baseline | ≥1 vs 0 | 2 vs 5 | 2.12 | 1.13-3.99 | .019 | 9 vs 3 | 2.26 | 1.20-4.23 | .011 |
